# Fluctuating environment causes neoplastic transition and epithelial-mesenchymal plasticity in human cells

**DOI:** 10.21203/rs.3.rs-10094171/v1

**Published:** 2026-07-31

**Authors:** Erez Persi, Rafael Canevarolo, Praneeth Reddy Sudalagunta, Liping Xu, Khadijeh Karbalaei, Gulden Olgun, Yuri Wolf, Sridhar Hannenhalli, Eugene Koonin, Ariosto Siqueira Silva

**Affiliations:** National Institutes of Health; H. Lee Moffitt Cancer Center & Research Institute; H. Lee Moffitt Cancer Center & Research Institute; Department of Metabolism and Physiology, H. Lee Moffitt Cancer Center & Research Institute; Stony Brook University; Department of Computer Engineering, Hacettepe University; National Institutes of Health; National Institutes of Health; National Institutes of Health; H Lee Moffitt Cancer Center and Research Institute

**Keywords:** Tumorigenesis, Tumor Evolution, Warburg Phenotype, Epithelial-Mesenchymal Plasticity (EMP), Genetic and Phenotypic Heterogeneity, Selection (dN/dS), Single Cell Multi-Omics, Breast Cancer

## Abstract

How environmental selective pressures contribute to the emergence of a neoplastic tumorigenic state, remains incompletely understood. Here we show that the immortalized non-tumorigenic breast epithelial cell line (MCF10A) develops neoplastic clones under fluctuating conditions in-vitro that mimic the harsh tumor microenvironment. Long-term evolutionary experiments and genomic landscape analyses of the clones identified ROS as the dominant clonal mutational signature that facilitates the acquisition of oncogenic driver mutations and the onset of the Warburg phenotype. Single cell multi-omics and brightfield microscopy further elucidate that each clone adopts two plastic phenotypic states, epithelial-like and mesenchymal-like, the transition between which is epigenetically mediated by de novo expression of Grainyhead-like Transcription Factor 2 (*GRHL2)*, following environmental cues from soluble factors and cell adhesion. Additional in-vitro experiments indicate the role of this plasticity in metastasis. Analysis of 70 human breast cancer cell lines and 2 independent breast cancer patient cohorts confirmed the generality of this mechanism and its epigenetic regulation in patients. Thus, this study elucidates fundamental evolutionary mechanisms behind tumorigenesis and identifies *GRHL2* as a potential therapeutic target against epithelial-mesenchymal plasticity and metastatic spread in breast cancer.

## Introduction

Carcinomas are the most common class of human cancers, and among these, breast cancer is one of the best-studied and frequently serves as a model system(1). Tumor initiation reflects the ability of mutated epithelial cells to overcome local physiological constraints, including spatial, metabolic, and signaling stresses, leading to the emergence of genetic and phenotypic heterogeneity characteristic of early carcinogenesis(2, 3). In the breast duct, loss of epithelial homeostasis results in hyperplastic expansion into the lumen, where cells become progressively isolated from the basement membrane and experience fluctuating access to oxygen, glucose, estrogen, and other growth signals. These variable microenvironmental pressures select for phenotypes that tolerate stress, including the Warburg Phenotype (WP), marked by lactate-producing aerobic glycolysis and microenvironmental acidification(4-6). These phenotypic changes promote genomic instability, impair antitumor immunity, and eventually, correlate with inferior clinical outcomes(7, 8).

To mimic these microenvironmental factors and elucidate how cancer cells acquire WP, we previously examined the growth of human breast tumorigenic cell lines (e.g., MCF7) under individual stresses such as hypoxia, acidosis, or nutrient deprivation. Although these conditions can select for adaptive mutations, such as loss of Tumor Protein p63 (*TP63*), and phenotypic traits, such as pan-therapy resistance and anchorage-independent growth, none of them alone resulted in WP emergence(9-11). In contrast, when cells were cultured under fluctuating conditions with episodes of hypoxia, acidosis, and nutrient deprivation, de novo selection of WP clones occurred(6). These findings suggest that the selective pressures driving WP must act concomitantly and repetitively, mirroring the fluctuating environments of early tumorigenesis(6, 12). However, it remains unknown, whether such fluctuating stresses can transform a non-tumorigenic epithelial cell into a neoplastic one, and how such pressures shape the genetic and epigenetic landscapes of an evolving, adapting cell population.

Temporal genomic analyses of patients and clonal single-cell studies have shown that most cancers consist of cells that have already accumulated multiple mutations by the time they are clinically detected(13, 14). Although the timing of mutation acquisition can be estimated(15), how these events relate to microenvironmental selective pressures, remains unclear. Beyond genomic changes, early-acting epigenetic mechanisms can generate phenotypes that are not predictable from mutational data alone(16-18). These epigenetic states are often heritable and can exert long-lasting effects on cellular behavior(19). Although both genetic and non-genetic processes in tumor evolution are well recognized, a mechanistic understanding of how the environment shapes these molecular landscapes is still lacking(20-23). Taken together, these observations raise a key question: how do microenvironmental fluctuating conditions induce or select epigenetic and genomic changes that jointly shape the evolutionary trajectory from pre-neoplastic cells toward cancer?

To address this question, we subjected the immortalized, non-tumorigenic human breast epithelial cell line MCF10A to fluctuating conditions that mimic the harsh tumor microenvironment. After two years of selection, individual clones were isolated by limiting dilution, expanded, and comprehensively profiled at the metabolic, genomic, transcriptomic, and epigenetic levels. We found that these clones evolved under intense oxidative stress, which promoted oncogenic events, and consistently exhibited the WP. Single-cell multiomics revealed that, despite marked genetic divergence, all clones converged toward a dynamic epithelial (**E**) – mesenchymal (**M**) plastic state, whereby the transitions between **E** and **M** states are epigenetically mediated via Grainyhead-like Transcription Factor 2 (GRHL2), a master regulator of epithelial cell development(24-26). To test the generality of this finding, we analyzed other breast cancer cell lines as well as two independent breast cancer patient cohorts, confirming the presence of this phenotypic convergence and its association with inferior clinical outcomes. We further provide experimental proof-of-concept demonstrating how this plasticity manifests in evolving populations to generate spatial **E-M** structures that could facilitate metastatic spread. Together, these findings indicate that microenvironmental fluctuations enable cellular evolvability and adaptability, and support a mechanistic model in which cancer initiation is accelerated by ROS mutagenesis, whereas cancer progression is marked by the emergence of WP and epithelial–mesenchymal plasticity (**EMP**). We propose targeting *GRHL2* as a therapeutic strategy against **EMP** to block metastatic spread.

## Results

### Long-term in-vitro culture of MCF10A cells under fluctuating conditions develops WP clones.

**Figure 1:**
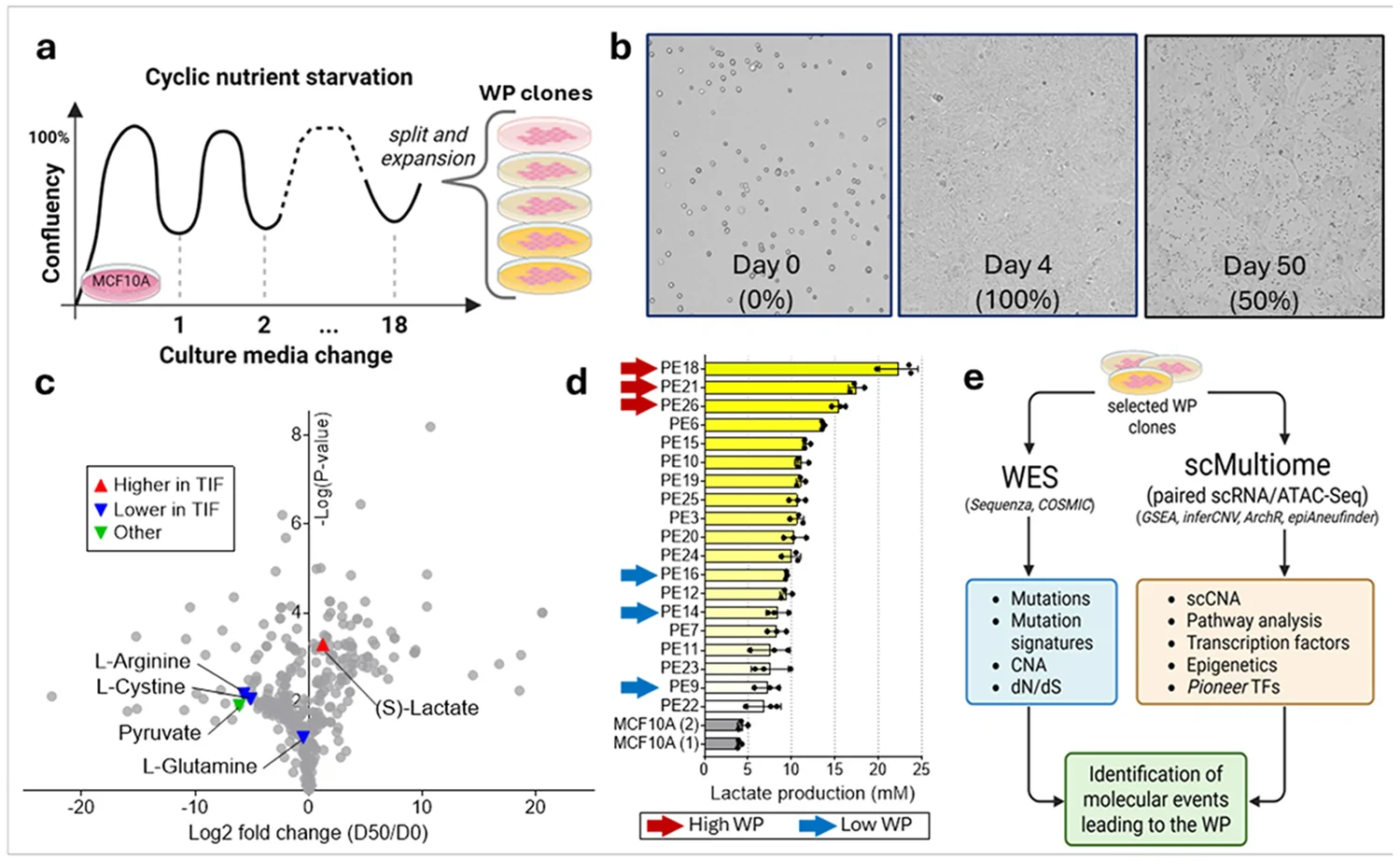
Experimental design and baseline characterization of cell clones. (**a**) Schematic of the cyclic starvation and recovery protocol used to generate Warburg-phenotype (WP) clones. (**b**) Microscopy images showing parental cell line progression from seeding to confluency to death (50% viability). (**c**) Volcano plot of metabolite differences in spent media at 50% viability, highlighting overlap with tumor interstitial fluid (TIF) metabolites. (**d**) Lactate production across isolated clones, with selected clones for downstream analysis highlighted. Data are presented as mean ± standard deviation (SD). (**e**) Overview of downstream profiling strategy, including WES and scMultiome.

We cultured the immortalized non-tumorigenic breast epithelial cell line (MCF10A) under conditions designed to reproduce in vitro the fluctuating microenvironmental selection pressures experienced in early breast carcinogenesis. The cells were allowed to grow in culture media, without passage or replenishment, until spontaneous cell death due to media depletion reduced viability below 50% of confluency, at which point, the spent media was collected for further analysis and replaced by fresh growth media, allowing cells to resume growth (Fig. 1a-b). After 2 years of such selection, clones were isolated and expanded from single cell colonies. Metabolomics analysis of the spent media (Fig. 1c) confirmed its similarity to tumor interstitial fluid (TIF), namely, lactate accumulation(27) and depletion of amino acids essential for ROS-metabolism, such as cystine(28), glutamine(29) and arginine(30). Aerobic lactate production, a WP hallmark, was measured (Fig. 1d), confirming that these clones produced significantly higher amounts of lactic acid aerobically compared to control. Six of these WP clones, 3 at the lower end and 3 at the higher end of the lactate production rate, hereafter denoted Low-WP and High-WP, respectively, along with one control clone, were isolated for further analysis. The clones displayed heterogeneous survival in spent media, whereby 2 out of 3 High-WP clones, and particularly PE26, exhibited reduced survival relative to parental cells (**Supplementary Fig. 1**). This finding suggests that the High-WP phenotype is associated with increased sensitivity to nutrient deprivation compared with Low-WP and parental cells. All 6 WP clones were characterized at the molecular level, including calling of point mutation and copy number alterations (CNA) from whole-exome sequencing (WES), and scMultiome (scRNA/ATAC-Seq), in order to assess single cell transcriptomic, epigenomic (chromatin accessibility) and CNA changes (Fig. 1e).

### Recurrent oncogenic events shared among clones and pre-clinical breast carcinogenesis models.

**Figure 2:**
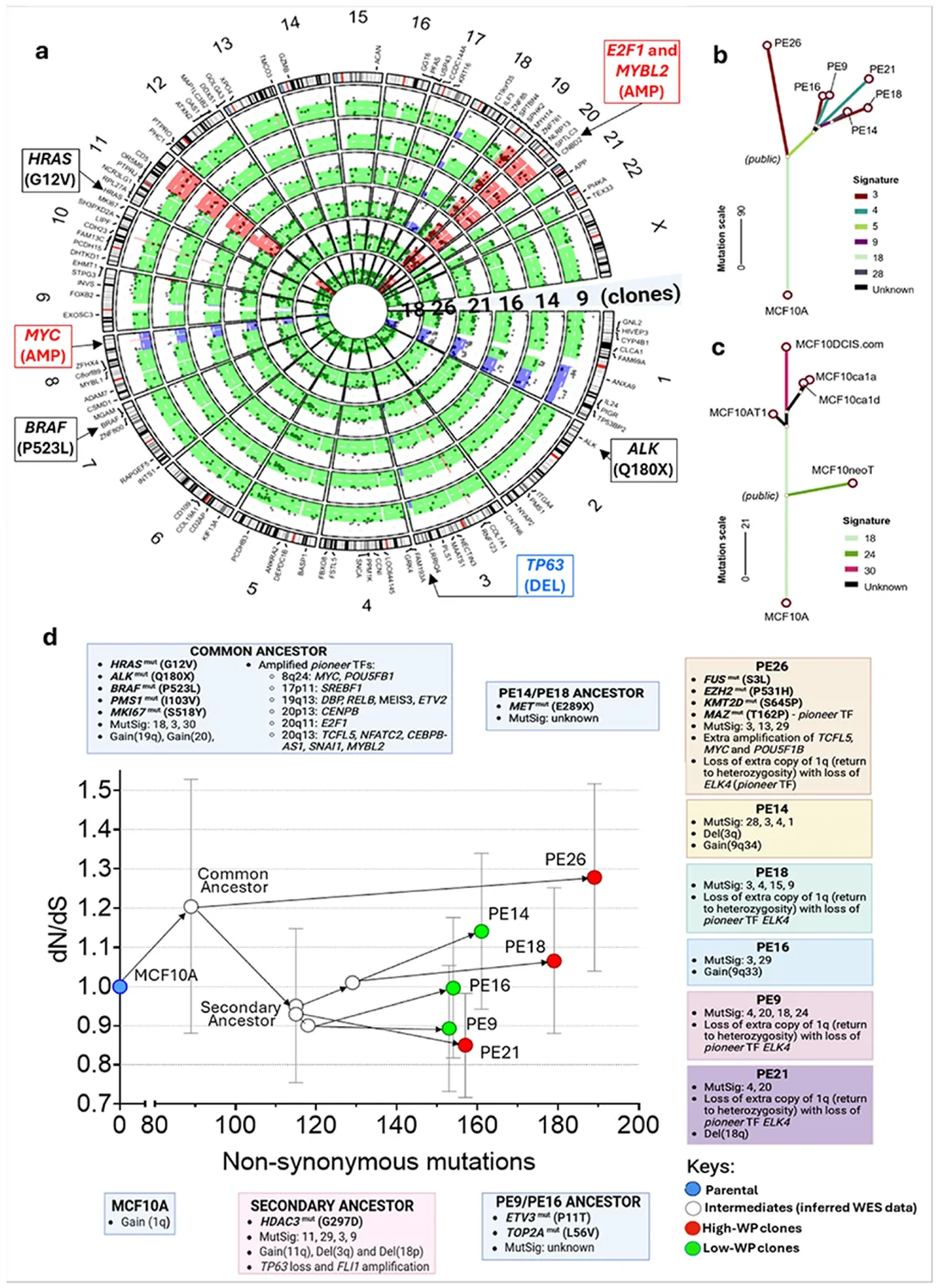
Genomic alterations and ROS mutagenesis signatures. (**a**) Mutations and copy number alterations (red = amplifications; blue = losses) across clones, including common oncogenic events (e.g., HRAS, BRAF, ALK). (**b**) Mutational signature trajectory reveals dominant ROS-associated COSMIC signature 18 in early ancestral clones, lost during progression to selected clones. (**c**) Parallel analysis of HRAS(G12V)/MCF10A transformation shows similar ROS-linked mutational patterns. (**d**) Phylogenetic tree and dN/dS values highlight the loss of selection efficacy under fluctuating environment, as clones evolve close to the neutral regime, with some variability in the dN/dS values across the clones. Error bars denote standard deviations around the mean in each clone from bootstrapping the mutation data 1000 times

WES of the 6 WP clones identified 340 unique non-synonymous mutations compared to the control MCF10A, 93 of which were shared by all 6 clones, including the oncogenic mutations, *HRAS* (G12V), *ALK* (Q180X), and *BRAF* (P523L) (Fig. 2a and **Supplementary Fig. 2**). Of the 93 shared mutations identified, 31 were also shared with a well-established family of tumorigenic *HRAS*-transformed MCF10A cell lines that were subsequently passaged extensively in animal models(31-33) (**Supplementary Fig. 3**). Given that this reference dataset contained 196 non-synonymous mutations against a common background of 7470 mutation calls, the observed overlap is significantly higher than expected by chance (12.7-fold enrichment; right tail *p* = 9.75 × 10^−27^, hypergeometric test; see Methods). suggesting a shared evolutionary trajectory in both settings and indicating that fluctuating environmental conditions alone suffice to select for oncogenic drivers that otherwise have to be transfected into cells to generate a tumorigenic state.

### Copy number analysis identifies gains of oncogenes and loss of tumor suppressor genes.

To assess copy number alterations in the clones, we conducted single-cell copy number analysis (scCNA) using scATAC-seq data (epiAneufinder(34)) to determine the baseline abnormalities of the parental MCF10A cells (**Supplementary Fig. 4a**). As shown previously(33), MCF10A carries amplifications in regions 1q (comprising the epidermal differentiation complex region(35)), 5q and 8q, in addition to smaller deletions in chromosomes 7, 15 and 19. The single cell resolution indicates intra-sample heterogeneity in the abnormalities 5q, 8q and 7. We further conducted scCNA analysis of the clones based on WES data (Sequenza(36)) using MCF10A as reference (Fig. 2a and **Supplementary Fig. 4b**). The common cytogenetic abnormalities found were the loss of 1q and 8q, thereby effectively reversing the original amplifications in MCF10A, amplification of 11q (except for PE26), loss of 18p (except for PE26, which was unchanged, and PE21, which lost the entire chromosome 18), as well as gains of chromosomes 19q and 20 (Fig. 2a and **Supplementary Fig. 4c**). In addition, focal CNAs were observed across the clones, such as *MYC, E2F1*, and *MYBL2* amplifications, as well as *TP63* deletion (Fig. 2a). Notably, 1q, 8q deletions, 19q amplification and *MYC* focal amplification have been also observed in the MCF10A *HRAS*-transformed lineages(33).

### Phylogenetic and mutational signature analyses suggest that early ROS exposure drives mutagenesis.

To identify possible drivers of mutagenesis in the emergence of WP, we conducted phylogenetic and mutational signature analyses (Fig. 2b). The mutational spectrum of the common ancestor was dominated by the COSMIC’s Signature 18, which is etiologically associated with ROS exposure(37). In contrast, the branches between the common ancestor and each WP clone shared no dominant signatures, suggesting repeated, independent adaptation to ROS (**Supplementary Fig. 5**). A similar analysis applied to the *HRAS*-transfected MCF10A lineage demonstrated similar dominance of ROS-related mutational signatures in the trunk of the phylogenetic tree (Fig. 2c)(37). Taken together, these results indicate that fluctuating environmental stress promotes the acquisition of oncogenic mutations in MCF10A cells through ROS-mediated mutagenesis. This is consistent with previous observations in Barrett's esophagus showing that cycles of hypoxia and reoxygenation generate bursts of ROS that increase mutation rates(38).

### Neutral drift defines the evolutionary regime of clones.

To investigate the evolutionary dynamics of clones under fluctuating conditions, we quantified protein-level selection strength using the genome-wide *dN/dS* ratio, that is, the ratio of the numbers of non-synonymous (*N*) to synonymous (*S*) mutations, normalized by the respective numbers of *N* and *S* sites in the genome (Fig. 2d). Neutral evolution (*dN/dS ≈ 1*) often dominates the evolution of tumors, whereas deviations from neutrality arise in early stages due to positive selection (*dN/dS > 1*) of drivers, and in late stages, due to purifying selection (*dN/dS < 1*) reflecting removal of accumulated deleterious passengers(23, 39-44). Neutrality has been shown to correlate with worse prognosis and thus corresponds to an evolutionary regime that maximizes tumor fitness, presumably, by ensuring the optimal balance between drivers and passengers, which enables cells to overcome selective pressures, including treatment(23, 42, 43, 45).

These observations were recapitulated in the present analysis. The trajectory from MCF10A to the common ancestor of the WP clones showed an elevated *dN/dS* ratio (*dN/dS = 1.2* ± 0.32). Although the significance of this elevated value is low, it likely reflects early fixation of ROS-induced drivers such as *HRAS, BRAF*, and *ALK* (Fig. 2d). The subsequent transition to the secondary ancestor showed a marked return to (near) neutrality (*dN/dS = 0.95 ± 0.2*), followed by divergence of the selection strengths across clones, around the neutral regime, with the mean *dN/dS = 1.04 ± 0.06* (Fig. 2d). This persistent neutrality apparently reflects the evolutionary regiome in fluctuating conditions, whereby the efficacy of selection is reduced and the differences between driver and passenger mutations are blurred, as predicted by theory(46). In this regime, the reduction in the efficacy of selection translates into variable *dN/dS* values across clones around the average neutrality. This mode of evolution leads to longer survival times of most clones compared to parental, except for clone PE26, which exhibited the highest *dN/dS* value (*dN/dS = 1.28 ± 0.24*), likely reflecting a lower fitness of this clone under continued stress (**Supplementary Fig. 1**).

### Single-cell transcriptomic analysis reveals phenotypic convergence toward EMP.

**Figure 3:**
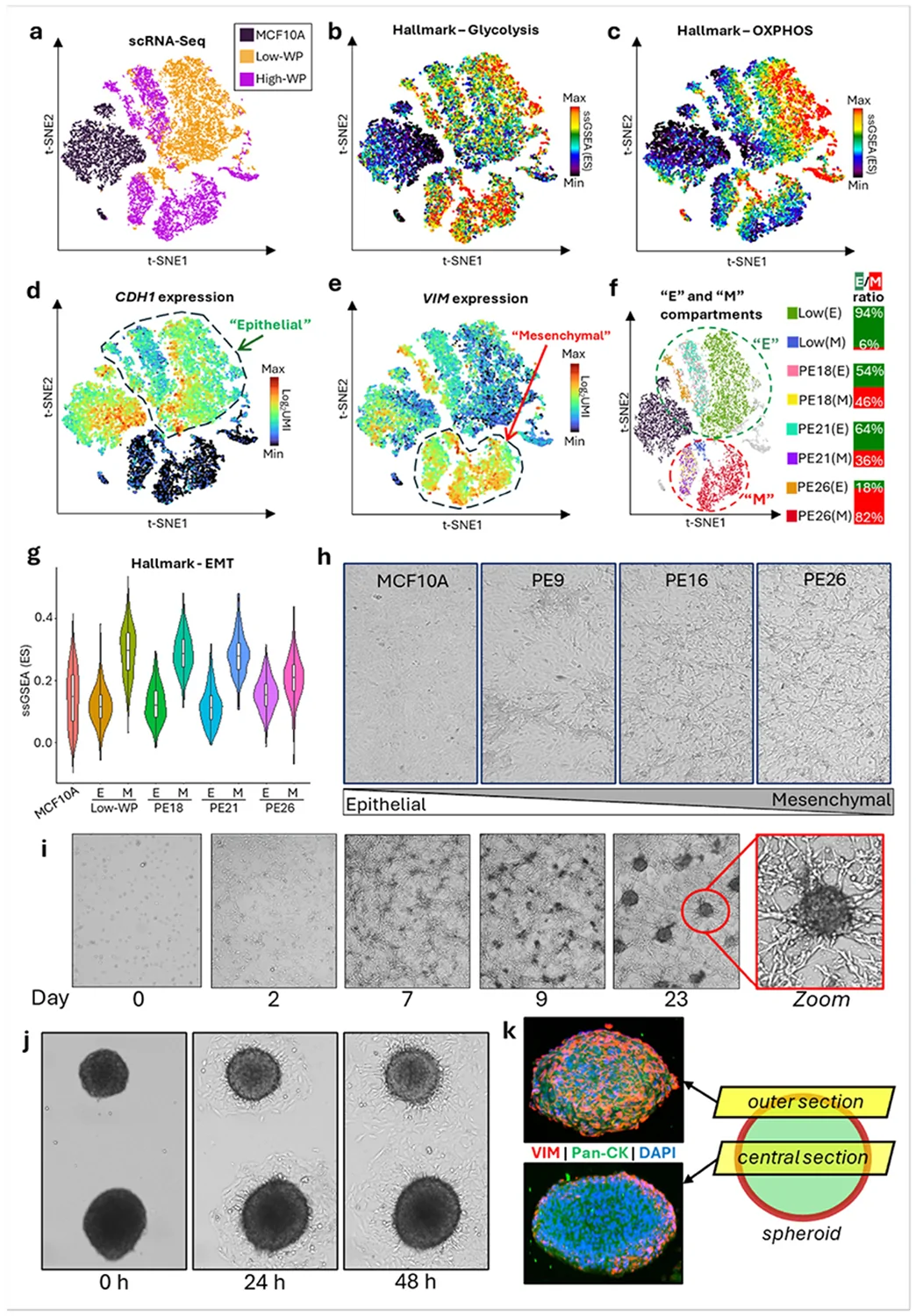
Single-Cell multiomes reveal metabolic profiles and EMP. (**a**) t-SNE of scRNA/scATAC profiles separates MCF10A, Low-WP, and High-WP groups, with overlap of some Low-WP cells into High-WP clusters. (**b-c**) High-WP cells, and to a lesser extent Low-WP cells show increased glycolysis and ROS transcriptional signatures. (**d-e**) Spatial expression maps of CDH1 and VIM across t-SNE space delineate epithelial and mesenchymal cell states. (**f**) Re-clustering of clones by **E/M** identity confirms subclonal heterogeneity. (**g**) ssGSEA scores of EMT programs reveal intra-clonal plasticity. (**h**) Morphological gradient mirrors EMT state. (**i**) Time-course of PE26 cells grown without extracellular matrix: single cells reach confluence by Day 2, form intertwined “knots” by Days 7–9, and compact into spheroids that later detach (Day 23). (**j**) Detached spheroids reattach and generate dense secondary spheroids. (**k**) Immunofluorescence of spheroid sections shows spatial EMT organization: peripheral layers express Vimentin (VIM, red), whereas the compact core displays increased pan-Cytokeratin (Pan-CK, green). DAPI (blue) marks nuclei. Schematic (right) indicates section positions.

Dimensionality reduction (t-SNE) and unsupervised clustering of scRNA-seq data from the six WP clones and parental MCF10A cells showed that parental cells formed a single cluster, whereas High-WP and Low-WP cells segregated into two groups of subclusters (Fig. 3a). Notably, a substantial number of High-WP cells mapped to the Low-WP clusters and vice versa, indicating marked intraclonal heterogeneity. To characterize the underlying biology underneath this heterogeneity, we performed single-cell ssGSEA, which confirmed increased glycolytic and oxidative phosphorylation pathway activity in WP clones relative to parental cells (Figs. 3b-c). Differential expression analysis between High- and Low-WP clusters identified two canonical **EMP** markers: E-cadherin (*CDH1*) was enriched in the Low-WP–dominated clusters (Log2 fold change = 5.025; adjusted *P* = 4.54 × 10^−39^, exact negative binomial test with Benjamini–Hochberg correction, see Methods) (Fig. 3d), whereas Vimentin (*VIM*) was enriched in the High-WP–dominated clusters (Log2 fold change= −2.787; adjusted *P* = 2.67 × 10^−28^) (Fig. 3e).

To further investigate state dynamics within individual clones, we integrated CNV information from WES (Sequenza) and scATAC-seq (epiAneufinder) to map individual clones onto the t-SNE space (**Supplementary Fig. 4d**). Low-WP clones lacked sufficient numbers of unique CNV events for individual discrimination and were therefore grouped as a single population, whereas High-WP clones could be resolved into **E** and **M** subsets. Cells from each clone appeared in both **E** and **M** regions, with High-WP clones predominantly in the **M**-state and Low-WP cells (combined) predominantly in the **E**-state (Fig. 3f), highlighting the difference between the high-WP and low-WP cells, but also confirming the transcriptional-level intraclonal heterogeneity, particularly with respect to **EMP** (Fig. 3g). Morphological analysis validated these transcriptomic observations: brightfield imaging revealed a progressive increase in mesenchymal morphology from parental to Low-WP to High-WP clones (Fig. 3h). Notably, the High-WP clone PE26, which evolved under positive selection, far from neutrality (*dN/dS* ≈ 1.3), consisted predominantly of cells in the **M**-state.

### Coexisting cells in E and M states in 2D culture rapidly self-organize into spheroid structures during continued growth.

Having established that WP clones contain intermixed cells in **E** and **M** states, we next asked how these populations behave when allowed to grow continuously in fresh media, mimicking well-vascularized regions of tumors. Under these conditions, parental MCF10A cells ceased proliferation, whereas in WP clones, particularly PE26, cells in the **M**-state continued to expand and spontaneously formed dense multicellular aggregates (“knots”) (Fig. 3i). These aggregates produced free-floating spheroids, detaching from the monolayer yet remaining viable in suspension. When transferred to a new flask, spheroids readily reattached and regenerated mixed **E-M** populations (Fig. 3j), indicating that the underlying **EMP** is maintained through serial transitions between adherent and suspension states. Immunofluorescence and confocal microscopy revealed a striking spatial structure within these spheroids: a compact **E**-state core enriched for pan-cytokeratin and *CDH1*, surrounded by an outer shell of VIM-positive **M**-state cells (Fig. 3k and **Supplementary Fig. 6**). This architecture implies rapid self-organization of **EMP** into a functional structure, in which an adhesive epithelial center is encased by a more motile and invasive mesenchymal layer. The speed with which this structure emerges, within a few days of spheroid formation, highlights the dynamic nature of the **EMP** in WP clones.

### Integrated multiomics analyses reveal GRHL2 as a key transcriptional and epigenetic mediator of EMP.

**Figure 4:**
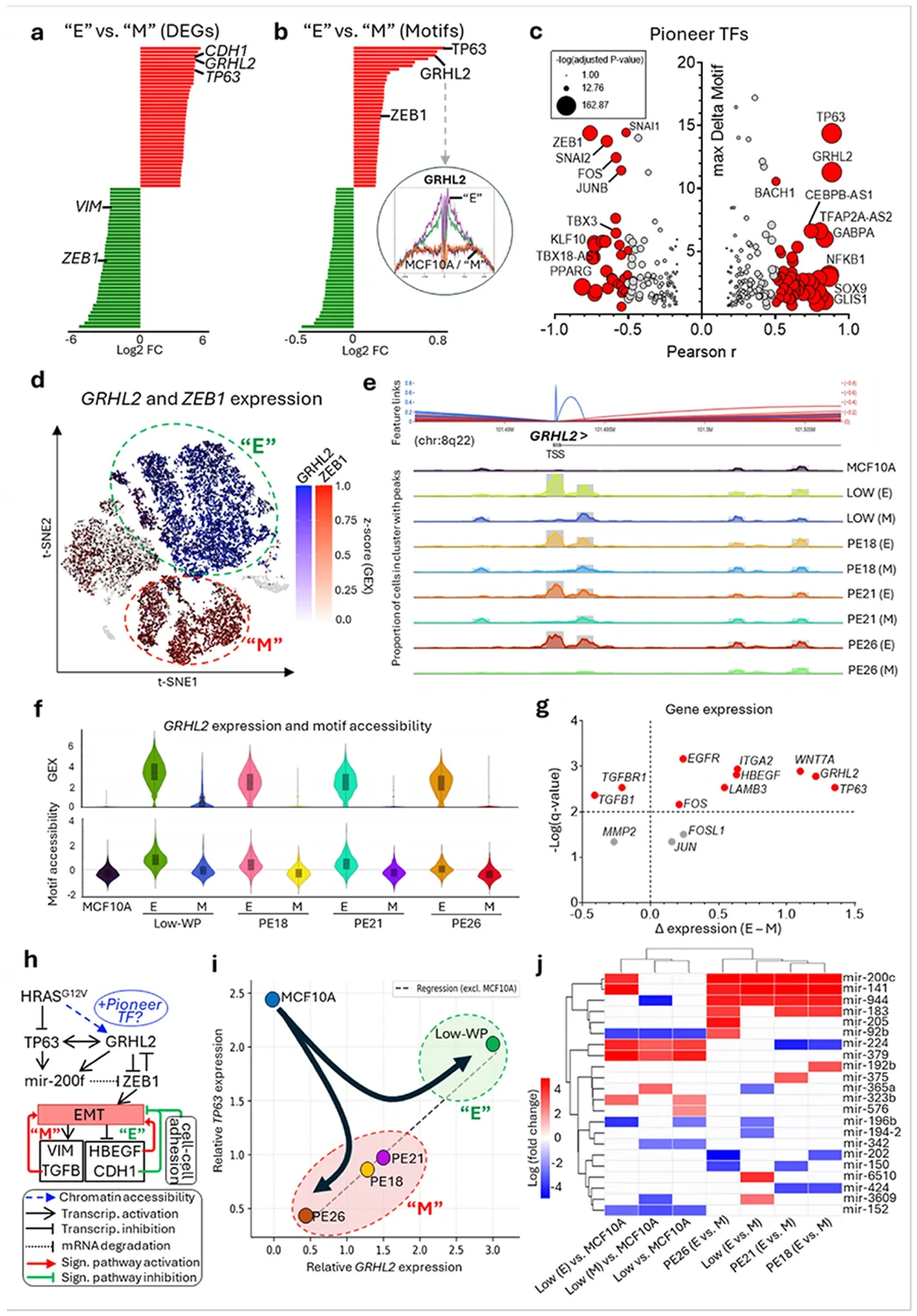
GRHL2 and TP63 define a transcriptional–epigenetic circuit governing EMP. (**a**) Unsupervised analysis reveals consistent differential expression of epithelial and mesenchymal markers between **E** and **M** subpopulations across different clones. Epithelial markers (CDH1, GRHL2, TP63) are enriched in **E**cells, while mesenchymal markers (VIM, ZEB1) are upregulated in **M** cells. (**b**) Motif accessibility analysis reveals that genes differentially expressed between **E** and **M**cells exhibit concordant changes in transcription factor (TF) binding site accessibility. Among the top-ranked motifs, TP63, GRHL2 and ZEB1 are more accessible in **E** cells. The inset highlights open chromatin surrounding GRHL2 motifs in **E** cells, in contrast to parental and **M** cells. (**c**) Volcano plot of TF expression–motif accessibility correlations identifies candidate pioneer DNA-binding proteins (DBPs). GRHL2, TP63, and ZEB1 display strong association between TF expression and target motif accessibility, supporting their putative roles as upstream regulators of **E/M**identity. GRHL2 lies in the upper-right quadrant, consistent with a pioneer-like role in promoting epithelial chromatin architecture. (**d**) UMAP projection of cells from multiple PE clones shows distinct epithelial and mesenchymal clusters, with GRHL2expression localized to **E** compartments and ZEB1 to **M** compartments, confirming spatial separation of cell states and corresponding regulators. (**e**) Chromatin accessibility tracks reveal that GRHL2’s own promoter is closed in parental and **M** cells but becomes open in **E** cells, suggesting a feed-forward epigenetic switch. (**f**) Violin plots show GRHL2 expression and accessibility of GRHL2 motifs across clones, supporting consistent activation of this axis in epithelial subpopulations. (**g**) Differential gene expression across **E** and **M** subpopulations in our scRNA-seq data, focusing on a curated list of genes identified by Sundqvist et al., 2020 as key effectors of collagen invasion in TP63-deficient MCF10A cells. Expression patterns in our dataset recapitulate this invasive signature, further supporting the functional relevance of the TP63–GRHL2 regulatory axis in controlling EMT and matrix interaction phenotypes. (**h**) Proposed model of **EMP** in WP clones: HRAS(G12V) regulates a core TP63–GRHL2–ZEB1–miR-200 network that governs transitions between **E** and **M** states. ZEB1 promotes EMT and represses GRHL2, whereas GRHL2 supports **E** identity through TP63 and miR-200, establishing a mutually antagonistic regulatory circuit. Cell–cell interactions further modulate this balance, enabling reversible transitions between **E** and **M**states (i) Phase-plane plot showing clone-specific expression of GRHL2 and TP63, relative to the parental line and fully epithelial or mesenchymal states. Clones occupy distinct positions along the **E↔M** axis, reflecting stable yet diverse epigenetic configurations. (**j**) Machine learning-based inference of regulatory activity identifies microRNAs (e.g., miR-200c) and TFs associated with differential **E/M** states across clones, reinforcing a multi-layered regulatory model of plasticity.

To characterize transcriptional programs underlying the **E** and **M** states, we performed differential gene expression analysis between the **E** and **M** clusters (Fig. 4a). As expected, **E**-state cells showed high *CDH1* and low *VIM* expression. The canonical epithelial regulator *TP63*, expressed in parental MCF10A cells, was enriched in the **E** clusters, whereas the mesenchymal master regulator Zinc Finger E-box Binding Homeobox 1 (*ZEB1*) was depleted. Notably, *GRHL2*, a *TP63* co-regulator of epithelial identity in several tissues(47, 48), was also strongly enriched in **E**-state cells. Given that **EMP**, mediated by *TP63–ZEB1*, is governed by chromatin remodeling(49, 50), we compared transcription factor (TF) motif accessibility across the **E** and **M** states using scATAC-seq. TP63 and GRHL2 motifs were among the top most differentially accessible motifs in **E**-state cells compared to **M**-state cells (Fig. 4b), consistent with activation of the respective downstream regulatory programs. Although *ZEB1* motifs also showed increased accessibility in **E**-state cells, this pattern aligned with reduced *ZEB1* activity because *ZEB1* functions primarily as a transcriptional repressor. Integrating transcriptomic and chromatin accessibility data, we sought to identify pioneer TFs shaping **EMP**. As expected, the **EMP** repressors *ZEB1* and *SNAI1* appeared as negative regulators of the chromatin accessibility of their targets, consistent with the demonstrated repression of Syndecan 1 (*SDC1*)(51) and *CDH1*(52, 53) by these TFs. In contrast, *TP63, GRHL2, BACH1*, and *NFKB1* emerged as activating pioneer TFs (Fig. 4c). We then investigated whether *GRHL2* participates in a negative feedback loop with *ZEB1*, as previously proposed in breast cancer models(54). Indeed, *GRHL2* and *ZEB1* displayed mutually exclusive expression in WP clones, with *GRHL2* expression undetectable in parental MCF10A cells (Fig. 4d). Consistently, analysis of chromatin accessibility showed that the *GRHL2* promoter is closed in parental cells but becomes accessible in **E**-state cells within each WP clone (Fig. 4e), and that *GRHL2* expression correlates with accessibility of its downstream targets (Fig. 4f).

### TGFβ and HBEGF paracrine loops contribute to the balance between E and M states.

Previous work on an *HRAS* (G12V)-transfected MCF10A model (MCF10AT1) has shown that breast cancer invasiveness requires both Transforming Growth Factor Beta (TGF-β) and Epidermal Growth Factor (EGF) signaling and depends on *TP63* expression(55). Moreover, parental MCF10A cells respond to TGFβ pulses by transiently switching from **E** to **M** states(56). Based on these observations, we hypothesized that the **E** and **M** states emerging in WP clones might be sustained by reciprocal paracrine signaling. Consistently, **M**-state cells of all WP clones expressed higher levels of TGFβ ligands than both the respective **E**-state cells and the parental MCF10A cells, whereas Heparin-Binding EGF-like Growth Factor (HBEGF) and EGFR were enriched in parental cells and **E**-state cells but depleted in **M**-state cells (Fig. 4g). Furthermore, genes downstream of the *EGF–TP63/GRHL2* axis, including *FOSL1, JUN*, and *FOS*, as well as collagen-invasion–associated genes (*ITGA2, LAMB3, WNT7A*), were preferentially expressed in the **E**-state cells (**Supplementary Fig. 7**), mirroring the *TP63*-dependent phenotype observed in the MCF10AT1 *TP63* knockdown model(55). In contrast, genes associated with TGFβ receptor signaling were consistently upregulated in the **M**-state cells (Fig. 4g).

### Proposed unified model of HRAS(G12V)-dependent EMP.

Integrating our findings with the *TP63*-knockdown MCF10AT1 synthetic model(55), we propose that **EMP** in WP clones is governed by two parallel *HRAS*(G12V)-driven transcriptional programs that converge on a core *GRHL2–TP63–ZEB1*–miR-200 regulatory axis (Fig. 4h). In MCF10A cells, *TP63* maintains epithelial identity by promoting expression of the miR-200 family of micro(mi)RNAs, which suppress *ZEB1* through mRNA degradation, thereby restraining epithelial-mesenchymal-transition (EMT). Under these conditions, *GRHL2* is epigenetically silenced, with promoter chromatin closed. Upon acquisition of activating *HRAS*(G12V) mutation, *TP63* is suppressed, whereas *GRHL2* is induced, consistent with prior reports(57). However, *HRAS*(G12V)- effect on *GRHL2* appears to be context-dependent because reduced expression of both *TP63* and *GRHL2* has also been observed in other MCF10A-based models(58), suggesting that additional upstream or clone-specific regulators might influence *GRHL2* accessibility and expression.

Mechanistically, suppression of *TP63* reduces transcription of miR-200(59, 60), a well-established post-transcriptional inhibitor of *ZEB1*. The loss of miR-200-mediated repression enables ZEB1 translation, which in turn represses *GRHL2*, highlighting the reciprocal antagonism between *ZEB1* and *GRHL2*(61), and promotes EMT through induction of mesenchymal markers, such as *VIM* and *TGFB*, as well as repression of epithelial-associated genes, including *CDH1* (**Supplementary Fig. 7**). We hypothesize that, in WP clones, additional genetic and/or cytogenetic alterations create a permissive epigenetic state in which the *GRHL2* promoter becomes accessible. Once expressed, *GRHL2* can act as a pioneer transcription factor that promotes *TP63* expression by binding to its promoter and increasing chromatin accessibility(59), consequently inducing the expression of miR-200(62). Through this pathway, *GRHL2* is able to partially restore the epithelial regulatory circuitry and oppose ZEB1-driven EMT.

Importantly, however, this rescue of the **E** state appears incomplete. Rather than re-establishing the stable **E** state observed in canonical MCF10A cells, *GRHL2* expression in WP clones induces a metastable configuration in which epithelial and mesenchymal programs remain in a dynamic balance. In this context, cell state becomes sensitive to environmental inputs. When cell density increases, enhanced cell-cell interactions and adhesion-associated signaling (e.g., through *CDH1*) suppress EMT programs(63), reduce *ZEB1* expression, and favor the **E** state. Conversely, when cell density decreases, reduced cell-cell interactions relieve this suppression and favor re-entry into the **M** state. More specifically, the **M** state is associated with high *TGFB* and *VIM* expression, whereas the **E** state is characterized by high *HBEGF* and *CDH1* expression. Thus, confluency maintains **E** cells in the **E** state and drives mesenchymal-epithelial-transition, whereas low cell density and pro-EMT cytokine signaling maintain **M** cells in the **M** state and promote EMT.

Together, these interactions define a bistable, environmentally responsive regulatory circuit in which *ZEB1* and *GRHL2* function as mutually antagonistic regulators of the cell state. *HRAS*(G12V)-mediated suppression of *TP63* biases the system toward a *ZEB1*-high **M** state, whereas clone-specific epigenetic activation of *GRHL2* provides a compensatory, albeit incomplete, epithelial rescue. The balance between EMT-inducing cytokines and epithelial-promoting cell-cell adhesion signaling determines the direction of transition, enabling **EMP** in WP clones. This dynamic relationship is captured quantitatively in the *TP63/GRHL2* phase plane, where individual clones occupy distinct yet continuous positions along the EM axis (Fig. 4i). In this framework, *HRAS*(G12V) functionally couples *TP63* and *GRHL2* expression, linking two regulatory axes that would otherwise remain uncoupled. Finally, because both *TP63* and *GRHL2* regulate *ZEB1* post-transcriptionally through miR-200(59, 62, 64), we investigated whether additional miRNAs might contribute to **EMP**. Using machine learning-based inference of miRNA activity from scRNA-seq data(65), we confirmed *miR-200c* as a dominant regulator, consistent with previous findings(47), and identified *miR-141*, which is encoded adjacent to *miR-200c* and is likely co-regulated with it(66, 67), as well as *miR-944*, located on a different chromosome and therefore potentially representing an independent modulatory mechanism (Fig. 4j).

### GRHL2 regulation of EMP is conserved across breast cancer cell lines.

**Figure 5:**
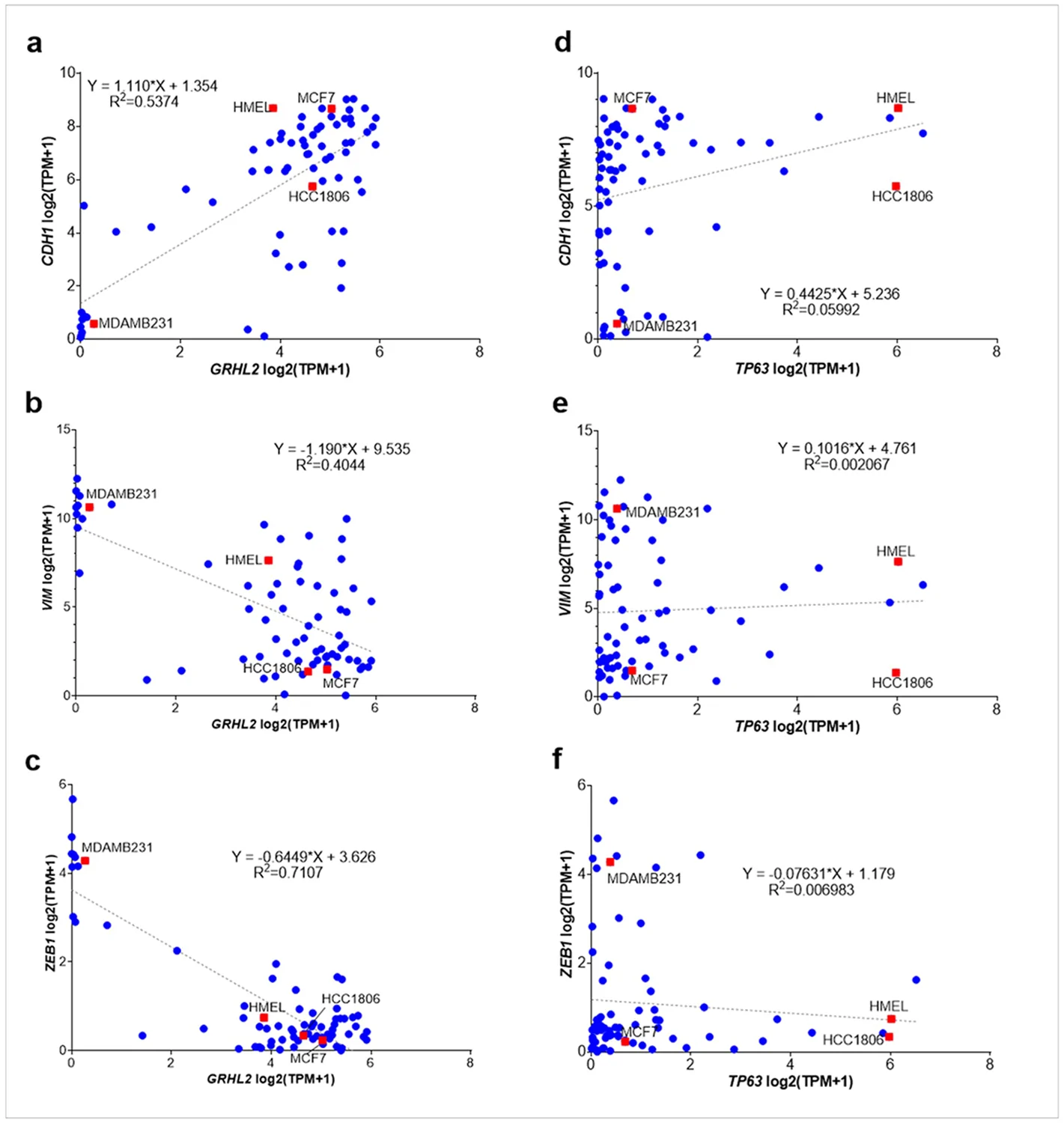
Expression of EMT marker genes correlates with *GRHL2* but not *TP63*in breast cancer cell lines. (**a–c**) Expression of *GRHL2* positively correlates with epithelial marker *CDH1* and negatively correlates with expression of mesenchymal marker *VIM* and EMT master regulator *ZEB1*in 70 human breast epithelial cell lines. These correlations are absent in *TP63*(**d-f**). Cell lines for which GRHL2’s role of EMT regulation was functionally validated(62, 70) have been labeled.

To determine whether the role of *GRHL2* observed in the WP clones is unique to MCF10A or conserved, we examined transcriptomic profiles from 70 breast cancer cell lines available through the DepMap Portal(68) (https://depmap.org/portal). These cell lines span a wide spectrum of genetic landscapes, allowing us to test whether the associations identified in the HRAS-mutant WP are conserved across cells. Across this diverse panel, *GRHL2* expression positively correlated with the epithelial marker *CDH1* and inversely with mesenchymal markers such as *VIM* and *ZEB1* (Fig. 5a-c). In contrast, *TP63* showed no consistent correlation with either epithelial or mesenchymal features (Fig. 5d-f), suggesting that, although *TP63* marks a stable epithelial baseline in some contexts, *GRHL2* is the variable regulator that modulates **EMP** in diverse breast cancer cell lines. Previous work has demonstrated that *HRAS* (G12V) transfected MCF10A cells increase *GRHL2* and reduce *TP63* expressions(57, 69) (**Supplementary Figs. 8a–b**), supporting a causal link between oncogenic *HRAS* signaling and the *GRHL2* transcriptional program.

Furthermore, in the human breast epithelium cell line HMLE, Tamoxifen-induced *TWIST*-mediated *GRHL2* suppression leads to EMT transcriptional profile (e.g. *CDH1* suppression), while *GRHL2* ectopic expression promotes a shift from a bi-modal **E-M** equilibrium to a purely **E**-state, while suppressing spheroid formation by **M** subpopulation (CD44^high^) (61, 62). Additionally, the same study demonstrated that ectopic expression of *GRHL2* in MDA-MB231-LN breast cancer cell line, induced mesenchymal to epithelial transition both morphologically and transcriptomically (e.g. expression of *CDH1*, suppression of *ZEB1*)(62). Independently, it was demonstrated that *GRHL2* suppression in two cell line models (HCC1806 and MCF7) shifts cell cycle towards G0/G1, decreases *in-vitro* growth, enhances 2D migration, as well as reduces primary tumor growth and metastases formation *in-vivo*(70). Together, these gain and loss of function findings indicate that **EMP** mediated by *GRHL2*, which we identified in the WP clones, is conserved across genetically diverse breast cancer cell lines.

### GRHL2 expression is associated with poor prognosis and is epigenetically regulated in breast cancer patients.

**Figure 6:**
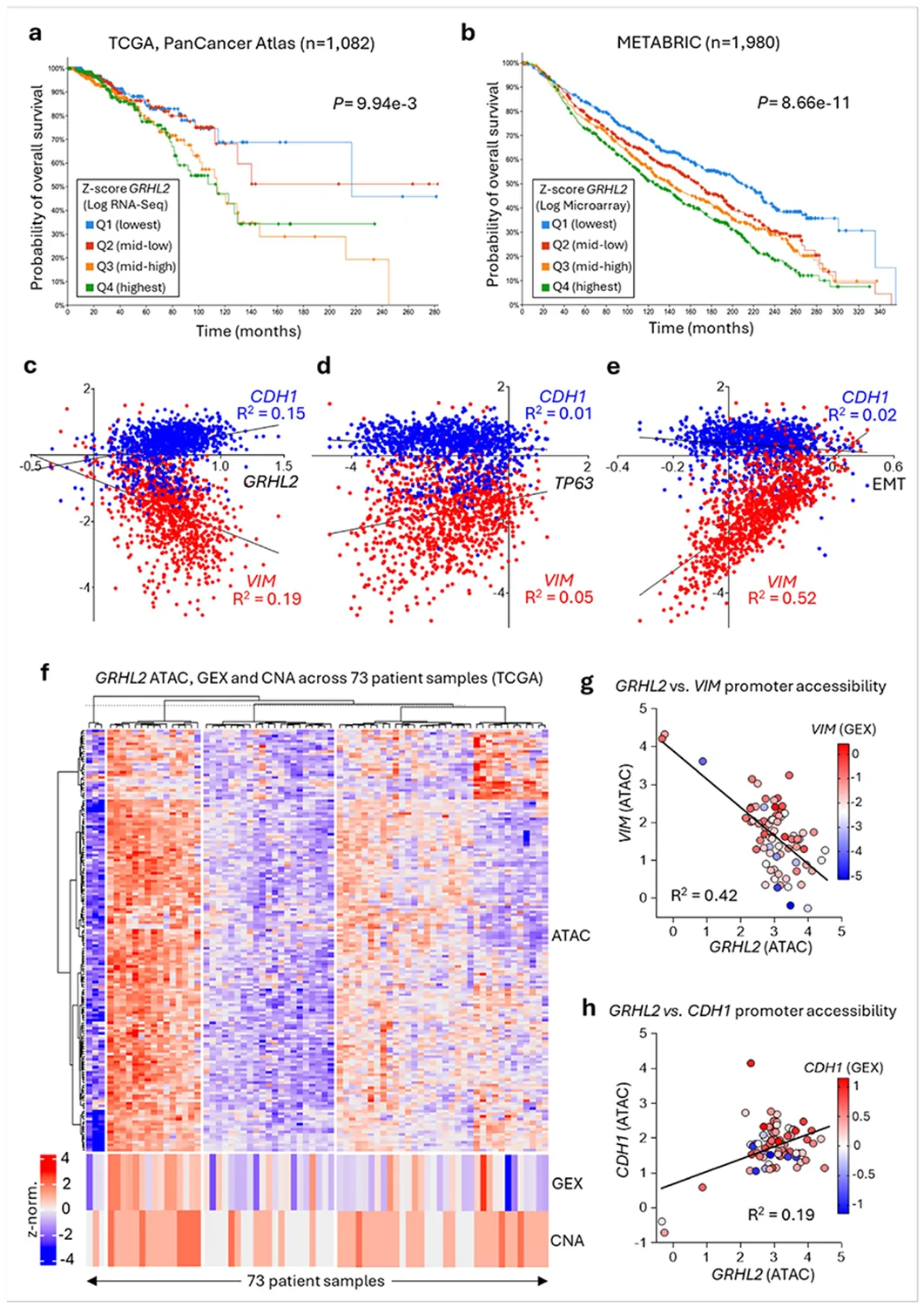
Epigenetic activation of GRHL2 correlates with EMT regulation and reduced survival in breast tumors. (**a–b**) Kaplan-Meier survival analysis in TCGA and METABRIC cohorts shows that high GRHL2 expression correlates with worse overall survival. (**c–e**) Scatterplots show GRHL2 and TP63 expression in breast tumors (TCGA), colored by VIM and CDH1, indicating that EMT marker expression correlates with GRHL2but not TP63. (**f**) ATAC-seq peak intensity across GRHL2-associated regulatory elements shows variable chromatin accessibility across tumor samples. GRHL2expression (top) correlates with accessibility, but not with copy number (bottom), confirming epigenetic regulation. (**g–h**) VIM and CDH1 promoter accessibility correlate with GRHL2 promoter accessibility, echoing the regulatory logic observed in cell line–derived clones.

Next, we tested whether the regulatory mechanism underlying EMP in breast cancer cell lines also operates in primary tumors of breast cancer patients. To address this question, we first investigated the prognostic significance of *GRHL2* expression in primary breast tumors by analyzing clinical and transcriptomic data from two large patient cohorts from cBioPortal: TCGA Breast Invasive Carcinoma (PanCancer Atlas, N = 1,082; RNA-seq) and METABRIC (N = 1,980; microarray). For each cohort, patients were stratified into quartiles according to *GRHL2* expression. Kaplan–Meier analysis showed that patients in the highest *GRHL2* quartile had significantly lower overall survival compared to those in the lowest quartile (log-rank p = 9.94 × 10^−3^ in TCGA; p = 8.66 × 10^−11^ in METABRIC) (Figs. 6a-b). Across the METABRIC cohort, higher *GRHL2* expression was associated with shorter overall survival time, both between and within molecular breast cancer subtypes, with the most pronounced effect observed in basal-like tumors (**Supplementary Fig. 9**).

Second, to determine whether *GRHL2*-mediated **EMP** extends to human tumors, we examined correlations between *GRHL2* and canonical markers in the PanCancer Atlas cohort. *CDH1* and *VIM* showed stronger associations with *GRHL2* expression (Fig. 6c) than with *TP63* (Fig. 6d), suggesting that *GRHL2*, rather than TP63, is the primary regulator of **EMP** in breast cancer (Fig. 6e). This observation parallels our in vitro findings, where *GRHL2* emerged not as an exclusive marker of either **E** or **M** cell state, but as a key mediator of the dynamic transition between those states.

To evaluate whether *GRHL2* regulation was epigenetically controlled also in patients, we analyzed a subset of samples with matched RNA-seq and ATAC-seq data(71). *GRHL2* expression was strongly associated with promoter accessibility, despite the heterogeneity of *GRHL2* copy number (Fig. 6f). Moreover, accessibility of the *GRHL2* promoter significantly correlated with both the expression and accessibility of promoters for the markers *CDH1* (Fig. 6g) and *VIM* (Fig. 6h). These findings support a conserved, epigenetically mediated regulatory architecture in which promoter accessibility governs *GRHL2* expression, which in turn modulates **EMP** in breast cancer. Together, these results establish GRHL2 as a clinically relevant, epigenetically regulated driver of **EMP**, with potential implications for prognosis and therapeutic stratification.

## Discussion

Previous studies have revealed extensive heterogeneity among cancer cells, but offer limited insight into how fluctuating, harsh tumor microenvironment shapes the genomic and epigenomic landscapes of tumors and the emergence of oncogenic phenotypes. Here, we address this by modeling how fluctuating environmental stress can drive the transformation of the non-tumorigenic cell line MCF10A into tumorigenic cells, yielding plastic phenotypes with therapeutic implications. Specifically, after 2 years of evolution under fluctuating environment, cells produced clones with hallmark features of breast carcinogenesis, including ROS mutagenesis(72), which promoted oncogenic genomic alterations, and adaptation in the form of WP(73) and **EMP**(74). These changes occurred without exogenous oncogene transduction, demonstrating that fluctuating environmental pressure is sufficient to induce and select for cancer hallmarks. We propose a model in which tumor-microenvironment stress induces new oncogenic mutations, such as *HRAS* (G12V) that, in turn, drive **EMP** by shifting the *GRHL2/TP63/ZEB1* regulatory balance, consistent with prior evidence from MCF10A cells engineered to express *HRAS* (G12V)(57, 69).

Strikingly, following divergence from a common ancestor, all WP clones converged to the same phenotype, with a bistable transcriptional state, whereby cells switch dynamically between **E** and **M** states. The **E**-state cells expressed *GRHL2* and *TP63*, exhibited open chromatin at epithelial loci, and repressed mesenchymal regulators such as *ZEB1*. In contrast, the **M**-state cells exhibited increased expression of *VIM* and closed chromatin at epithelial gene sites. These reversible states form a bistable **EMP** regulated by *GRHL2*, which is absent in parental MCF10A but is epigenetically activated in WP clones. *GRHL2* expression correlates with expression of soluble factors, including TGF-β and EGF (negatively and positively, respectively), both likely regulated by upstream TFs such as *FOS* and *KLF4*, and both upregulated in WP clones(6, 75, 76). This reversible program recapitulates the **EMP** dynamics predicted by theoretical models(77), and supports previous observations of hybrid **E-M** states in aggressive tumors(59, 78).

The *GRHL2*-mediated **EMP** is not limited to this model system. Analysis of 70 breast cancer cell lines from the DepMap Portal confirmed the correlation between the expression of *GRHL2* (but not *TP63*) and epithelial and mesenchymal gene markers, *CDH1* and *VIM*, respectively. Further, transcriptomic data from PanCancer Atlas and METABRIC cohorts demonstrated that high *GRHL2* expression correlated with its promoter accessibility and with poor prognosis in breast cancer patients. Thus, *GRHL2*-mediated **EMP** is widespread in breast cancer. This epigenetically regulated **EMP** might contribute to metastatic potential via the formation of circulating spheroids, a mechanism consistent with previous reports linking *GRHL2* to anoikis resistance and ROS detoxification in mammary epithelial cells(70, 79), and observed in WP clones cultured in fresh (continuously replenished) media. Previously, this plastic phenotype was comprehensively studied in colorectal cancer (CRC)(80), leading to the conclusion that most of the intratumor genetic variation in CRC had no major phenotypic consequences, whereas transcriptional plasticity was widespread within tumors. Thus, these findings in CRC are consistent with our interpretation that the genomic alterations are important for tumor fitness, especially, early in tumorigenesis, endowing cells with a selective advantage. In later stages, the emergence of **EMP** via an epigenetic mechanism confers adaptation to fluctuating environmental conditions and is likely critical for metastatic spread. We show here that *GRHL2* functions as a gatekeeper of this plasticity, integrating environmental cues into a reversible state-switch that confers survival advantage under fluctuating conditions(12).

**Figure 7:**
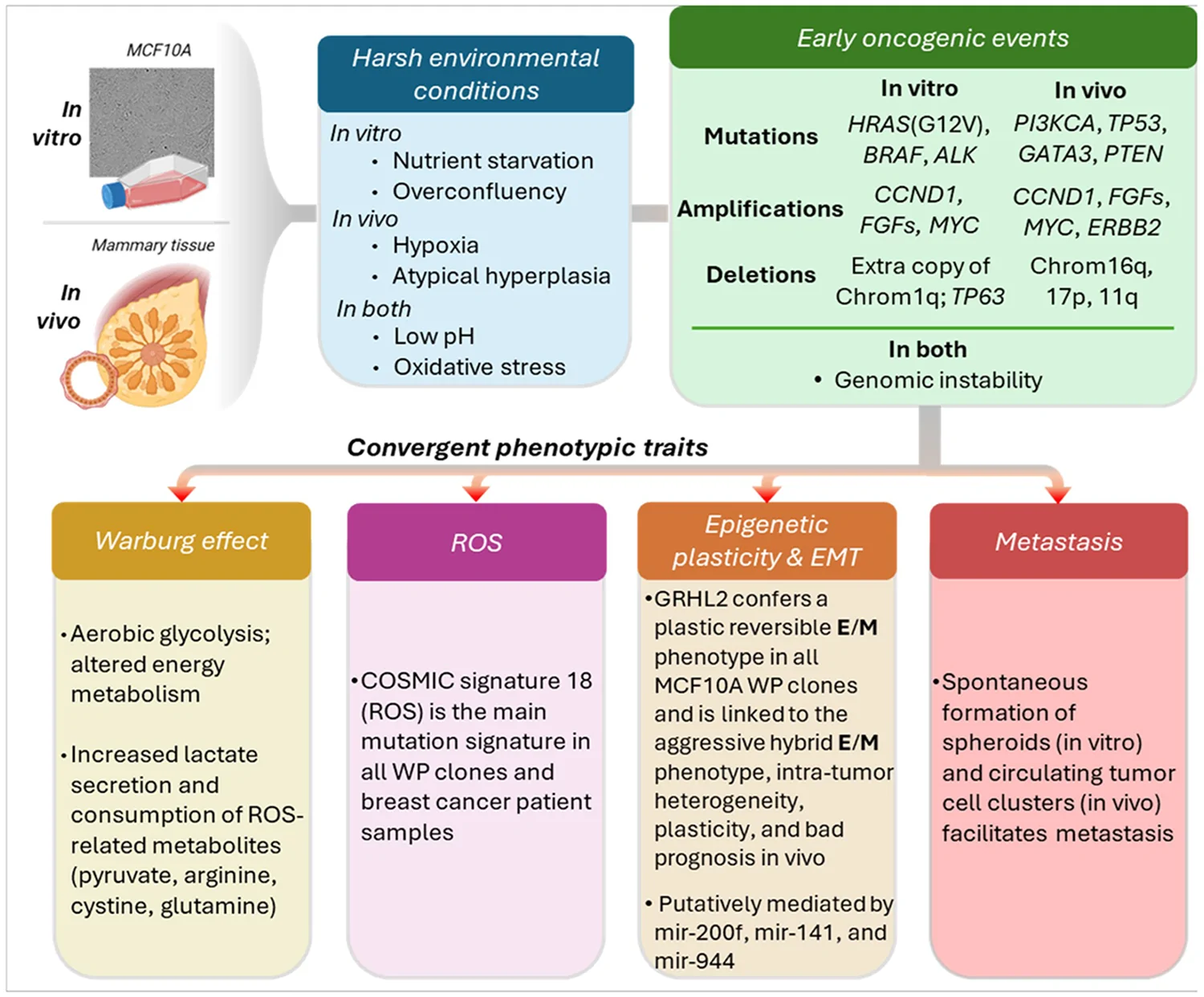
Model of early oncogenic events. Schematic model summarizing the convergence of environmental stress and genetic alterations in driving cellular transformation. Harsh in-vitro conditions—including low pH, oxidative stress, nutrient deprivation, hypoxia, and over confluency—mimic microenvironmental pressures present in-vivo, leading to atypical morphologies and survival adaptations. Independently, early oncogenic events such as mutations, amplifications, and deletions (categorized by their in-vitro, in-vivo, or shared contexts) promote genomic instability and sustained proliferation. Despite distinct genetic landscapes, both stress-induced and mutation-driven transformation converge on common phenotypic programs: Warburg metabolism, ROS accumulation, epithelial–mesenchymal plasticity, and metastatic competence. Central to this convergence is the regulation of GRHL2, a transcriptional gatekeeper of epithelial identity and plasticity, observed as a shared mechanism across MCF10A-derived stress models and human breast tumors.

Across our evolutionary experiments, fluctuating microenvironmental conditions consistently selected for clones exhibiting metabolic rewiring, oxidative stress tolerance, and a *GRHL2*-mediated **EMP**. We show that this plasticity is not limited to 2D cultures: WP clones rapidly self-organized into spheroids with a mesenchymal outer shell and an epithelial core (*cf.* Figure 3k). This spatial architecture is characteristic of coordinated collective migration of tumor cells, as observed in previous *in-vivo* studies demonstrating that multicellular spheroids (or circulating tumor cell clusters) possessed substantially higher metastatic capacity than single cells(81). Thus, the ability of WP clones to generate spheroids with spatial structure characteristic of **EMP** provides a mechanistic framework linking *GRHL2*-mediated plasticity to the inferior overall survival observed in high *GRHL2* breast cancers. Figure 7 integrates these findings, summarizing how environmental selection pressures converge on a phenotype characterized by metabolic adaptation, ROS tolerance, *GRHL2*-mediated plasticity, and the emergence of multicellular structures with enhanced metastatic potential, despite the apparently different genetic landscapes observed between cell line models and patients (e.g., *HRAS* mutations in the former and *PIK3C*, in the latter).

We propose that the **EMP** can be exploited therapeutically, particularly, by targeting the reversible **E-M** states that underpin metastatic competence. Agents that reinforce epithelial identity, such as TGFβ pathway inhibitors, *HDAC* or *LSD1* inhibitors, or miR-200 mimics, could limit metastatic potential by stabilizing the **E** state and suppressing transitions into the motile, invasive **M** state. Conversely, therapies known to preferentially target mesenchymal populations, including eribulin or emerging ferroptosis-based strategies, could eliminate the more invasive **M** compartment(82, 83). Together, these options suggest a **E-M** cell–specific therapeutic logic: either locking cells into a less invasive epithelial program or selectively eliminating mesenchymal subpopulations. Our results further imply that temporal coordination, matching therapy to the current phenotypic state of the tumor, will be essential. Because **EMP** is rapid and reversible, treatment regimens that ignore state dynamics could miss the therapeutic window, whereas “state-aware” or sequential **EMP**-directed strategies potentially would better constrain progression and metastatic dissemination.

In summary, long-term exposure of human cell cultures to fluctuating environmental conditions that mimic the harsh tumor microenvironment, lead to cancer evolvability and adaptability, reminiscent of similar observations on bacterial evolution(84), demonstrating how a normal cell transforms into a neoplastic one via mutagenesis, and how the progeny population adapts to fluctuating conditions via phenotypic convergence to **EMP**, which is epigenetically mediated by a druggable activation of *GRHL2*. Thus, this study not only unravels fundamental mechanisms of cancer initiation and progression, but also translates these into an opportunity for therapeutic intervention in breast cancer, by targeting *GRHL2* to inhibit **EMP**.

## Methods

### Selection of MFC10A WP clones

MCF10A cells were cultured in T75 flasks with 15 mL of DMEM/F12 medium supplemented with EGF (10 ng/mL), hydrocortisone (0.5 μg/mL), cholera toxin (100 ng/mL), insulin (5 μg/mL), 5% horse serum, and 1% penicillin–streptomycin. Cells were initially seeded at approximately 25% confluency and allowed to grow to full confluency. Cultures were maintained without passaging until spontaneous cell death reduced confluency to below 50%, as monitored by microscopy at regular intervals. At this point, the medium was replaced without trypsinization, allowing the remaining cells to recover and repopulate the culture. This cyclic process of growth, spontaneous cell death, and recovery was repeated over a period of two years. Individual clones were subsequently isolated by limiting dilution in 96-well plates and expanded for downstream analyses. The parental MCF10A line and all derived WP clones were tested for mycoplasma contamination and authenticated in accordance with ATCC guidelines.

### Lactate production measurements

Cells were seeded in 24-well plates in growth medium containing 10% FBS under standard culture conditions. Upon reaching approximately 90% confluency, the growth medium was removed, and cells were washed twice with phosphate-buffered saline (PBS) before incubation in phenol red–free medium supplemented with 2% FBS for 24 hours. Conditioned media were then collected for lactate quantification. L(+)-lactate levels were measured using a colorimetric assay kit (BioVision, catalog #K627-100) according to the manufacturer’s instructions. Absorbance was measured at 450 nm and background-corrected using medium (2% FBS) from wells without cells. Lactate production rates were normalized to total protein content per well. Lactate and glucose concentrations were also independently measured using a YSI 2900 Multi-Analyte System (YSI, Yellow Springs, OH), following the manufacturer’s protocol.

### Whole exome sequencing and mutational analysis

Whole-exome sequencing (WES) was performed on parental MCF10A cells and the six WP clones to identify somatic mutations in coding regions of the genome. Genomic DNA (200 ng per sample) was processed using the Agilent SureSelect XT Clinical Research Exome kit, which includes the targets of the Agilent v5 whole-exome kit with enhanced coverage of ~ 5,000 disease-associated genes (Agilent Technologies, Santa Clara, CA). Library preparation was performed according to the manufacturer’s protocol, and library quality and fragment size distribution were assessed using an Agilent BioAnalyzer.

Equimolar amounts of library DNA were subjected to exome capture using Agilent capture baits. Following enrichment, libraries were quantified by qPCR and quality-checked on the BioAnalyzer prior to sequencing. Approximately 150 million paired-end reads (75 bp) per sample were generated using v2 chemistry on an Illumina NextSeq 500 platform (Illumina, Inc., San Diego, CA).

Sequencing reads were aligned to the GRCh38.d1.vd1 reference genome using BWA (v0.7.17). Somatic mutation calling was performed using a consensus approach requiring agreement from at least two out of five variant callers for single-nucleotide variants (SNVs; FreeBayes, SomaticSniper, Strelka1, MuSE, and MuTect1) and insertion/deletions (INDELs; SomaticIndelDetector, FreeBayes, Strelka1, Strelka2, and MuTect2). Parental MCF10A cells were used as the reference for mutation calling. Variant call format (VCF) and mutation annotation format (MAF) files were generated, with the MAF file provided as **Supplementary Table S1**.

Phylogenetic relationships among samples were reconstructed using MesKit(85) (v1.6). Mutations shared across all WP clones, together with copy number information, were visualized using circular plots generated with the *circlize* R package (v0.4.15).

Mutational signature analysis was performed using MesKit(85) (v1.6) based on COSMIC v2 signatures. Signatures were inferred separately for trunk and branch mutations in the phylogenetic tree to identify potential drivers of mutagenesis across the parental line, a putative common WP ancestor, and derived WP clones.

### Estimating the significance of mutation overlap using hypergeometric test

Mutation overlap significance was assessed in MATLAB. The set of 93 (K) mutations shared across all 6 WP clones and the set of 196 (n) mutations identified in the *HRAS*-transformed reference dataset were treated as draws from a common background universe of size 7470 (N) mutation calls (total mutation calls from Maguire et al.(33)), and the observed overlap was 31 (x). The expected overlap under random sampling was calculated as (K×n)/N and fold enrichment was computed as x/[(K×n)/N]. To avoid numerical underflow for extremely small probabilities, hypergeometric probabilities were not computed directly with *hygepdf* or *hygecdf*, but instead were calculated in log-space using the gamma-function identity for binomial coefficients implemented with MATLAB’s gammaln function: gammaln(a + 1) - gammaln(b + 1) - gammaln(a-b + 1). The right-tailed hypergeometric p-value, P(X ≥ x), was computed by evaluating the log-probability for each overlap value from x:min(K,n) and summing these terms using m = max(log_terms); log_p_right = m + log(sum(exp(log_terms - m))).

#### Copy number analysis from WES data

Copy number alterations (CNAs) were inferred from WES data using Sequenza(36) (v3.0) from aligned BAM files, with parental MCF10A cells used as the reference sample. Analyses were performed using default parameters. Sequenza-derived segmentation identified genomic regions with altered copy numbers, which were reported both as relative fold changes compared with the parental reference and as estimated absolute copy number states. CNA results are provided in **Supplementary Table S2**.

##### Selection ( dN/dS ) analysis

Two main approaches have been developed in recent years to estimate the selection force at the molecular level, known as the *dN/dS* ratio(86), the ratio between the rate of non-synonymous (*N*) mutations and the rate of synonymous (*S*) mutations, either at the gene level(40) or at the genome level(23, 42, 43). In both cases, the main limiting factor is attaining sufficient statistics of the number of *N* and *S* mutations, for accurate estimation of the *dN/dS* ratio. At the gene level, this can be attained by integrating the number of mutations across many patients and samples. This assumes that the physiological and environmental diversity across patients and samples can be neglected and emphasizes the importance of gene identity and function. At the genome level, sufficient statistics can be attained by integrating the number of mutations in the genome (i.e., across genes). This assumes that the differences among genes can be neglected and emphasizes the importance of physiology and environment in each sample and patient, in determining the average value of selection strength acting on the tumor genome. Both approaches capture the dominance of neutral evolution, consistent with other estimates(41, 44).

Here, because of the low number of samples (i.e., clones), we measure the *dN/dS* ratio at the genome level, due to statistical considerations. Further, our previous results of cell lines evolving under stress(6), as well as the current study, highlight the importance of environment in determining the course of evolution of these cells, rendering the approach of estimating *dN/dS* at the genome level particularly relevant. Briefly, the method treats the genome as a single concatenated sequence and calculates the number of non-synonymous and synonymous sites across the human genomes to appropriately normalize the number of *N* and *S* mutations in WES data. Statistical errors (*cf.* Figure 2d) were estimated by bootstrapping the number of mutations in each clone, simulating sampling with replacement of *N* and *S* mutations 1000 times and re-estimating *dN/dS* ratios. For further information see(23, 42, 43).

### Single-cell paired ATAC/RNA sequencing and analysis

Sample processing and library preparation were performed following the 10x Genomics Chromium Single Cell Multiome protocols (CG000365 and CG000338). Briefly, cryopreserved cells were thawed and gradually resuspended in pre-warmed culture medium. Cells were centrifuged at 500 × *g* for 5 minutes at room temperature, resuspended in warm medium, and viability was assessed using trypan blue staining (Countess; Thermo Fisher Scientific). For nuclei isolation, cells were pelleted and lysed in cold lysis buffer (0.1×) containing digitonin (0.15×). Lysis progression was monitored in real time (every 3 minutes) by trypan blue staining until cell viability dropped below 5% (typically 6–10 minutes). Nuclei were then washed three times in cold wash buffer, resuspended in nuclei buffer, and filtered through a 40 μm Flowmi cell strainer (SP Bel-Art).

Following transposition, nuclei were immediately loaded onto the Chromium Single Cell Controller (10x Genomics) using the Chromium Next GEM Chip J. Transposed nuclei, reagents, and barcoded gel beads were partitioned into Gel Beads-in-Emulsion (GEMs), where linear amplification and barcoding were performed. Libraries were subsequently recovered and amplified using the Chromium Next GEM Single Cell ATAC Reagent Kit v1.1 (10x Genomics). Sequencing was performed on an Illumina NextSeq 500 platform, generating approximately 25,000 (ATAC) and 50,000 (RNA) read pairs per cell, according to the manufacturer’s instructions.

Due to cost constraints, scMultiome profiling was performed on three samples: (i) parental MCF10A cells, (ii) a pooled sample comprising the three Low-WP clones, and (iii) a pooled sample comprising the three High-WP clones. Whole-exome sequencing (WES)-derived copy number alteration (CNA) profiles were used to assign cells to individual WP clones within pooled samples. Raw data were processed using Cell Ranger ARC (10x Genomics), and libraries were aggregated using the *aggr* function to generate a unified, normalized dataset across all samples. Loupe Browser (v8.0; 10x Genomics) was used for visualization, cluster segmentation, and exploration of chromatin accessibility and gene expression profiles across genomic regions.

Differential expression analysis was performed using Loupe Browser, which dynamically applies the Cell Ranger statistical framework. Gene expression differences between selected groups of cells were assessed using an exact negative binomial test based on the sSeq method(87), with asymptotic approximation for large counts, and p-values were adjusted using the Benjamini–Hochberg method. Chromatin accessibility tracks were exported from Loupe Browser in BED format and visualized using the UCSC Genome Browser (GRCh38/hg38). Cluster annotations were exported and incorporated into downstream analyses using ArchR (v1.0.2). Functional enrichment analysis was performed using the Enrichr(88-90) R package (v3.2).

### Single-cell copy number analysis

Single-cell copy number alterations (CNAs) were inferred using two complementary approaches: inferCNV(91) (v1.12.0) applied to scRNA-seq data, and epiAneufinder(34) (v0.1.0) applied to scATAC-seq data. Each method has inherent strengths and limitations, reflecting the properties of their underlying data modalities (e.g., scRNA-seq–based inference is restricted to expressed regions, whereas scATAC-seq–based CNA calls may be confounded by changes in chromatin accessibility). Integration of these single-cell CNA profiles with WES-derived CNA data enabled the identification of distinct cellular clusters within each of the three High-WP clones. In contrast, Low-WP clones exhibited highly similar CNA profiles by WES and lacked clear separation in scMultiome UMAP/t-SNE embeddings; therefore, they were analyzed as a single group for downstream single-cell analyses.

### miRNA activity estimation

miRSCAPE(65) uses supervised machine-learning to estimate miRNA activity from transcriptome-wide mRNA expression data, in either bulk and single-cell levels. Here, we trained the miRSCAPE model based on paired mRNA–miRNA data from The Cancer Genome Atlas (TCGA) breast cancer samples. The genes that were expressed in at least 1% of the cells in our single cell samples were used as features to train the miRSCAPE model. We then applied the trained miRSCAPE model to each scRNA-seq sample separately; to improve robustness, cells were aggregated into meta-cells by randomly pooling 100 cells, repeated 1,000 times. For each miRNA, in each sample, we thus predict 1000 bootstrapped activities of the miRNA. Predicted miRNA activities were then compared across conditions to identify differentially active miRNAs in each condition using the limma R package(92).

Results were summarized in a heatmap generated in R using the *pheatmap* package. For each comparison, the top eight miRNAs ranked by absolute log fold-change were selected. To standardize directionality across contrasts, log fold-change values were sign-corrected where needed, missing values were set to zero, and rows or columns with zero variance were removed prior to clustering. Extreme values were truncated at the 95th percentile of the absolute value distribution to improve visualization. Hierarchical clustering was performed using maximum distance for both rows and columns and complete linkage.

### Preparation of PE26 spheroid sections and immunofluorescence staining

PE26 cells were cultured in T75 flasks containing 15 mL of DMEM/F12 medium (Gibco, catalog #11320-033) supplemented with 10% heat-inactivated fetal bovine serum (FBS; Biowest, catalog #S1480), 1% penicillin–streptomycin (Gibco, catalog #15140122), hydrocortisone (0.5 μg/mL; Sigma-Aldrich, catalog #H-4001), cholera toxin (100 ng/mL; Sigma-Aldrich, catalog #C8052), human epidermal growth factor (hEGF; 20 ng/mL; R&D Systems, catalog #236-EG-200), and insulin (10 μg/mL; Sigma-Aldrich, catalog #I9278). Culture medium was replaced three times per week (Monday, Wednesday, and Friday). Under these conditions, floating spheroids typically emerged after approximately 2–3 weeks in culture.

Floating spheroids were collected from the supernatant and centrifuged at 300 × *g* for 1 minute. The resulting pellet was fixed in 10% neutral-buffered formalin (NBF) at 4°C for 5 hours and subsequently washed three times with phosphate-buffered saline (PBS). Spheroids were embedded in approximately 3 mL of 6.5% low-melting-point agarose (catalog # BP160-100, Fisher Scientific) using the bulb of a plastic transfer pipette as a mold and allowed to solidify at 4°C for 5 minutes. The solidified agarose block containing the spheroids was mounted on a Leica VT1200S vibratome in cold 1× PBS and sectioned at a speed of 1 mm/s and amplitude of 0.4 mm, with slices of 100 μm thickness between cuts (**Supplementary Fig. 10** shows representative images of the experimental setup and the spheroid–agarose block). Sections were collected on positively charged slides and stored at 4°C until staining.

For Pan-CK/Vimentin/DAPI immunofluorescence staining, sections were permeabilized with 0.1% Triton X-100 for 15 minutes, followed by blocking in 1% bovine serum albumin (BSA) in PBS. Samples were incubated for 1 hour at room temperature with fluorescently conjugated antibodies: anti-Pan-Cytokeratin (clone AE1 + AE3, Alexa Fluor 532, Novus, catalog # NBP2-33200) and anti-Vimentin (clone E-5, Alexa Fluor 647, Santa Cruz, catalog # sc-373717 AF647). Nuclei were counterstained with DAPI.

For E-cadherin/Vimentin/DAPI staining, spheroids were permeabilized with 0.5% Triton X-100 in PBS for 30 minutes, then blocked for 2 hours in 1% BSA/TBS/0.25% Triton X-100. Sections were incubated overnight at 4°C with primary antibodies prepared in blocking buffer, followed by incubation with the appropriate fluorescent secondary antibodies in blocking buffer for 2–3 hours at room temperature. The E-cadherin antibody used was a rabbit FITC-conjugated antibody (FabGennix International Inc., catalog # ECAD.131-FITC), raised against multiple synthetic peptides within amino acid residues 150–700 of human E-cadherin.

Fluorescence imaging was performed using a Leica Stellaris confocal microscope (Leica Microsystems GmbH, Wetzlar, Germany). Spheroids stained with DAPI, Alexa 488, and Alexa 647 were excited with 405 nm, 488 nm, and 647 nm laser lines through 2.5×/0.07 NA, 5×/0.15 NA, 10×/0.4 NA, 16×/0.6 NA, and 20×/0.75 NA objective lenses. Emissions were captured sequentially with three HyD S detectors tuned to the appropriate wavelengths for each fluorophore. Z-stack images were acquired using system-optimized slice thickness, and volume renderings were generated with the 3D module within LAS X software (v4.8.1.29271, Leica Microsystems GmbH). All system parameters, including laser power, gain, and offset, were kept constant across all samples within each magnification. Images were exported in TIFF format with embedded scale bars and processed using LAS X and Fiji (ImageJ) software for visualization and quantification.

## Supplementary Material

This is a list of supplementary files associated with this preprint. Click to download.

- TableS1SNP.xlsx
- TableS2CNV.xlsx
- CancerEvolvabilitySuppInfo.docx
